# Evaluation of the anti-fatigue effects of a traditional herbal drug, *Gongjin*-*dan*, under insufficient sleep conditions: study protocol for a randomised controlled trial

**DOI:** 10.1186/s13063-016-1542-7

**Published:** 2016-08-22

**Authors:** Mi Ju Son, Hwi-Jin Im, Young-Eun Kim, Boncho Ku, Jun-Hwan Lee, Chang-Gue Son

**Affiliations:** 1 Clinical Research Division, Korea Institute of Oriental Medicine, 1672 Yuseongdae-ro, Yuseong-gu, Daejeon, 34054 Republic of Korea; 2 Liver and Immunology Research Center, Daejeon Korean Medicine Hospital of Daejeon University, 176-9, Daeheung-ro, Jung-gu, Daejeon, 34929 Republic of Korea; 3 Mibyeong Research Center, Korea Institute of Oriental Medicine, 1672 Yuseongdae-ro, Yuseong-gu, Daejeon, 34054 Republic of Korea; 4 KM Fundamental Research Division, Korea Institute of Oriental Medicine, 1672 Yuseongdae-ro, Yuseong-gu, Daejeon, 34054 Republic of Korea; 5 Korean Medicine Life Science, University of Science & Technology, Campus of Korea Institute of Oriental Medicine, 1672 Yuseongdae-ro, Yuseong-gu, Daejeon, Republic of Korea

**Keywords:** Fatigue, Traditional herbal medicine, Randomised controlled trial, Protocol

## Abstract

**Background:**

Many herbal medicines are traditionally used as anti-fatigue agents in east Asian countries; however, there is a dearth of clinical evidence supporting the anti-fatigue effects of such medicines and their mechanisms. This study is a feasibility trial to assess the clinical efficacy of *Gongjin*-*dan* (GJD) and verify its mechanisms by exploring fatigue outcomes, including endocrine and immunological biomarkers in humans.

**Methods/Design:**

To investigate the anti-fatigue effects of GJD and the mechanism underlying these effects, a randomised, double-blind, placebo-controlled crossover clinical trial was designed. Participants (24 healthy male volunteers) will be hospitalised for 4 days (3 nights), during which acute fatigue and stress conditions will be induced by sleep deprivation, and GJD or a placebo will be administered (twice daily). The primary outcome will be changes in serum cortisol levels, measured in the morning, as an objective biomarker of sleep deprivation-induced fatigue and stress. The secondary outcomes will include: the Fatigue Severity Scale; the Brief Fatigue Inventory, and the Leeds Sleep Evaluation Questionnaire scores; levels of salivary cortisol, epinephrine, norepinephrine, oxidative stress-related biomarkers, homocysteine, and immunological factors; and heart rate variability. After a washout period of more than 4 weeks, a second treatment phase will commence in which participants who were previously administered the placebo will receive the drug and vice versa, following the same treatment regime as in the first phase.

**Discussion:**

This study protocol provides a unique opportunity to enhance our understanding of fatigue and the effects of GJD on fatigue in terms of endocrine and immunological mechanisms by validating the study design and determining feasibility. Findings from this trial will help researchers to design a pilot or definitive clinical trial of traditional herbal medicine for chronic fatigue.

**Trial registration:**

Korean National Clinical Trial Registry CRIS; KCT0001681, registered on 29 October 2015.

## Background

Fatigue is a subjective symptom of lethargy experienced during or after performing daily work, or a feeling of a lack of energy sufficient to affect daily life. The average worldwide incidence of chronic fatigue is 11.1 % and most fatigue resolves with rest; however, treatment is required when fatigue is prolonged and reduces the quality of life of individuals and their families, or generally affects society [1, 2]. Among people with chronic fatigue, labor force productivity is known to decrease by 54 % and the cost of lost productivity is estimated to be $9.1 billion annually in the US. [3].

Fatigue is a multifactorial condition and the pathophysiological mechanisms of idiopathic chronic fatigue or chronic fatigue syndrome are unclear. For this reason, there is no standard treatment for chronic fatigue [4] and some patients with chronic fatigue, therefore, turn to complementary and alternative medicine (CAM) for treatment [5, 6]. In East Asian countries, CAM in the form of traditional herbal medicine is widely used to treat fatigue [7, 8]; the traditional herbal drug, *Gongjin*-*dan* (GJD), is generally prescribed by Korean traditional medicine physicians to treat patients with chronic fatigue in Korean traditional medicine clinics. Although the anti-fatigue effects of herbal medicines, including GJD [9], deer antler extract [10], and *tao*-*hong*-*si*-*we*-*tang* [11], have been demonstrated in animal studies, few clinical studies have focussed on these treatments. To encourage evidence-based practices in the traditional medicine field, well-designed studies must be conducted to evaluate the effects and mechanisms of action of traditional herbal medicines, including traditional anti-fatigue medicines.

This study is a feasibility trial to assess the clinical efficacy of GJD and verify its mechanisms by exploring fatigue outcomes, including endocrine and immunological biomarkers. The unique protocol described herein is designed to minimise confounding factors and other possible bias, and comprises a randomised, double-blinded, placebo-controlled crossover clinical trial with hospitalised subjects. However, the findings from this trial cannot be generalised to individuals suffering with chronic fatigue syndrome because sleep-deprived healthy men aged 19–45 years were chosen as the participants of this study to avoid confounding factors and improve internal validity.

This study design may serve as a guide for researchers seeking to effectively evaluate the effects and underlying mechanisms of traditional herbal anti-fatigue medicines as well as conventional drugs.

## Methods

### Objective

The aim of this study was to develop a protocol to (1) evaluate the anti-fatigue effects of GJD in acute fatigue; (2) verify the mechanisms underlying the observed effects by assessing changes in stress hormones, oxidative stress-related biomarkers, homocysteine, and immunological factors; and (3) clinically assess the effectiveness and safety of GJD in treating acute fatigue using the Korean version of the Fatigue Severity Scale (FSS-K), Brief Fatigue Inventory (BFI-K), and Leeds Sleep Evaluation Questionnaire (KMLSEQ) scores as well as carrying out daily fatigue and sleep evaluations and assessments of heart rate variability (HRV) and adverse events.

### Design and setting

This randomised, double-blind (both patient and practitioner or assessor), placebo-controlled crossover clinical trial will be conducted in Republic of Korea.

#### Recruitment period

Participant recruitment began in September 2015 at the Daejeon Korean Medicine Hospital of Daejeon University, and it is expected to be completed in December 2015.

#### Methods of recruitment

Twenty-four participants will be recruited for this trial, and participants will be recruited using online and printed advertisement of the study. Advertisements will be posted on the online bulletin board of the Daejeon Korean Medicine Hospital of Daejeon University, and printed advertisements will be posted in stores in university towns and on school bulletin boards. Advertisements will also be posted on online social networking platforms. The relevant trial information will be conveyed to the potential participants prior to their first visit to the hospital.

#### Study plan

Subject information will be collected after oral and written consent is obtained from each participant at the first visit.

##### Screening phase

After potential participants voluntarily consent to the study, they will be screened using pre-determined inclusion/exclusion criteria at the first visit. The participants will be instructed to sleep for at least 7 h daily for 1 week before admission and to avoid new medications, alcohol, and caffeine consumption for 2 days before admission. Participants will be randomly allocated to one of two groups. The group allocation will be concealed from both the participants and the practitioners.

##### Treatment and evaluation phase

**Fig. 1:**
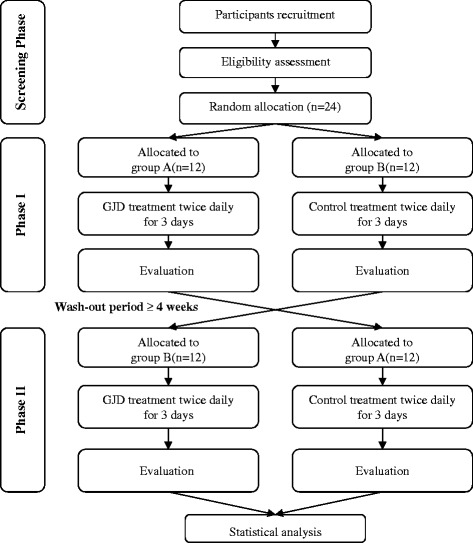
Flowchart describing the study plan

Both the treatment and placebo-control groups will be hospitalised for 4 days, and after a washout period of over 4 weeks the participants will be hospitalised again for 4 days. During hospitalisation, subjects will be administered GJD or placebo (one pill twice a day 30 min before breakfast and dinner) for 3 days. Group A participants will be administered GJD in phase I, followed by placebo treatment in phase II (both treatments will be administered twice daily for 3 days each). Group B participants will be administered placebo in phase I, followed by GJD treatment in phase II (same regimen as in group A; Fig. 1). To assess the effects of GJD treatment, participants will be allowed to sleep for only 4 h (02:00 to 06:00) on days 2 and 3 to artificially induce fatigue, and will carry out various activities such as walking, taking lectures, and watching movies to limit daytime sleep. To control possible bias, all subjects will be limited to intensive exercise and will consume the same foods during the study periods.

Blood samples will be collected at 06:30 for analysis of serum cortisol, stress hormones, oxidative stress-related biomarkers, homocysteine, and immunological factors. Saliva samples will be collected at 06:30, 12:00, and 21:00 for analysis of salivary cortisol, while HRV will be assessed at 15:00–17:00. Self-reporting of FSS-K, BFI-K, KMLSEQ, and daily fatigue and sleep status will be carried out via questionnaires after dinner on days 1, 2, 3, and 4.

After a 4-week washout period, participants will be admitted to phase II for 4 days, during which they will undergo the same schedule.

**Fig. 2:**
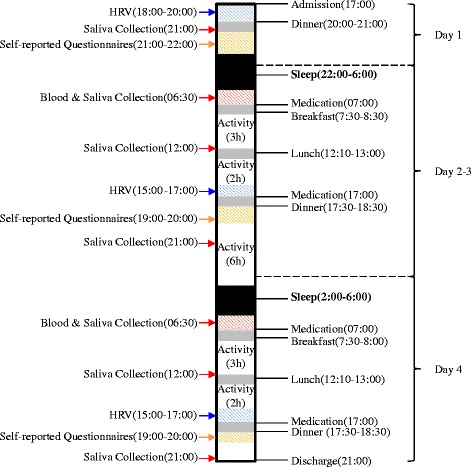
Timeline of participant activities, treatments, and assessments during each study phase

The schedule of events for the trial is outlined in Table 1 and the study participants’ timeline of activities, treatments, and assessments during hospitalisation is shown in Fig. 2.

**Table 1:**
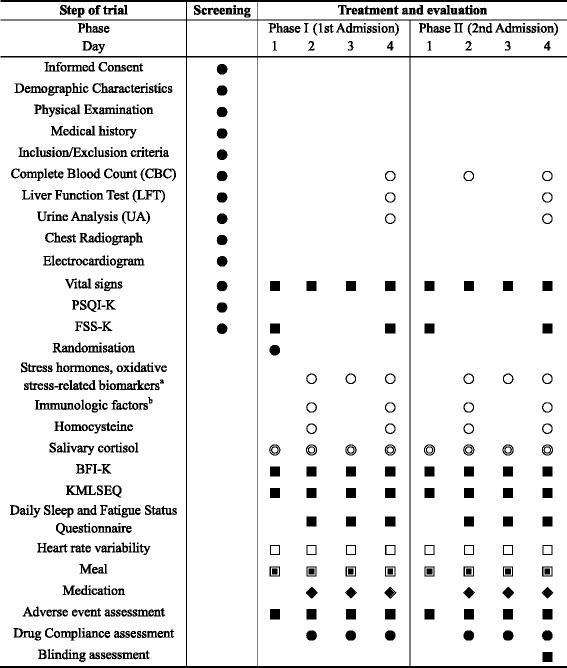
Schedule of treatments and outcome measurements

^a^Stress hormones; oxidative stress-related biomarkers: serum cortisol, epinephrine, norepinephrine; ROS, NO, MDA, protein carbonyl, GSH, GSH-Rx, SOD, catalase, TAC

^b^Immunological factors: TNF-α, IFN-γ, IL-2, IL-10, IL-12, T cell, B cell, NK cell

● Evaluation at any time; ○ 06:30 (30 min after waking up); ◎ 06:30 (30 min after waking up), 12:00 (before lunch), and 21:00; □ 15:00–17:00 (before dinner); ▣ 07:30–08:30, 12:10–13:00, and 17:30–18:30; ■ 18:30–21:00 (after dinner); ◈ 1 pill twice a day, 30 min before breakfast/dinner

### Study participants

#### Inclusion criteria

Participants are eligible for inclusion in the study if they meet the following criteria:

1. Healthy male volunteers aged 19 to 45 years at the screening visit
2. Participants must weigh over 45 kg, with a body mass index of 18.5–30 kg/m^2^ at the screening visit
3. Participants must not have any congenital defects or chronic diseases that have been present within the 3 years prior to the screening visit and should have no pathological or abnormal clinical findings on physical examination
4. Participants must not have any sleep disorders and must have Pittsburgh Sleep Quality Index (Korean version; PSQI-K) scores of 5 points or lower
5. Participant must keep normal nocturnal sleeping hours (between 21:00 and 09:00) for a week before the first administration of the investigational drug (or placebo)
6. Participants must sleep at least 7 h per night for a week before the first administration of the investigational drug (or placebo)
7. Participants must be nonsmokers or ex-smokers who stopped smoking at least 1 year before the screening visit
8. Participants must comprehend the purpose and process of the study as well as the properties of the investigational drug and must voluntarily sign a written informed consent approved by the Institutional Review Board of Daejeon Korean Medicine Hospital of Daejeon University

#### Exclusion criteria

Participants who experience, have, or have had one or more of the following will be excluded:

1. A clinically significant medical history or a current disease in one of the following areas: cardiovascular, respiratory, gastrointestinal, hepatic, metabolic and endocrine, renal and urinary, reproductive, musculoskeletal, skin and connective tissue, neurological, psychiatric, or allergic (other than seasonal allergies that are asymptomatic at the screening visit) disease
2. A medical history of gastrointestinal diseases (oesophageal achalasia, oesophageal stricture, or Crohn’s disease) or operations (other than simple appendectomy or hernia surgeries) that may affect drug absorption
3. Laboratory test results showing alanine aminotransferase (ALT) or aspartate aminotransferase (AST) levels of more than twice the upper limit of the normal range
4. A history of alcohol or drug abuse within the 12 months before the screening visit
5. History of regular alcohol consumption (>210 g/week) within the 6 months before the screening visit. (Beer (5 % v/v): 250 mL = 10 g, *soju* (20 % v/v): 50 mL = 8 g, wine (12 % v/v): 125 mL = 12 g)
6. Participation in another clinical study during the 2 months before the screening visit
7. Treatment with drugs with known effects on drug-metabolising enzymes (e.g. dexamethasone, phenytoin, carbamazepine, rifabutin, rifampicin, phenobarbital, ketoconazole, itraconazole, clarithromycin, atazanavir, indinavir, nelfinavir, ritonavir, clarithromycin, telithromycin, voriconazole, etc.) during the 30 days before the screening visit
8. Whole blood donation during the 2 months before the screening visit, or specific blood component donation in the month before the screening visit
9. Seated systolic blood pressure ≥140 mmHg or diastolic blood pressure ≥90 mmHg at the screening visit
10.  Intake of prescribed or over-the-counter drugs during the 7 days before the first administration of the investigational drug (or placebo)
11.  Consumption of alcohol during the 7 days before the first administration of the investigational drug (or placebo)
12.  Serious, acute, or chronic medical or mental conditions or abnormal laboratory findings that may be exacerbated by administration of the investigational drug or participation in the study, or that may affect the study outcomes
13.  Hypersensitivity to the investigational drug or any of its components
14.  Absence of intention or ability to adhere to the behavioural rules and requirements as specified in the present protocol
15.  Any person considered unsuitable for participation in the study by the principal investigator

### Intervention

Trial medications will be prepared by Kyoung-Bang Pharmaceutical Co. Ltd. (Incheon, Republic of Korea) certified in Good Manufacturing Practices of herbal medicine extract pills and granules from the Ministry of Food and Drug Safety (MFDS). Quality control and quality assurance on the quality and safety testing, the packaging, and the contents of the treatment interventions including checks for potential contaminants, such as heavy metals or steroids, will be undertaken by the manufacturers to ensure treatment drug stability and quality.

Quality control and quality assurance regarding the identification of products as well as quality and safety testing will be conducted by the manufacturer. The raw herbs will be washed, dried, ground, and mixed with a pure form of honey (*Mel*) before being wrapped in gold foil and formed into pills. The pills will be packaged in identically shaped containers weighing 3.75 g each. The GJD and placebo pills will be formulated to a similar size, shape, taste, and flavour and will be wrapped in gold foil.

GJD consists of three medicinal herbs (*Ginseng radix*, Korean angelica, *Corni fructus*), two animal-derived materials (*Cornus cervi parvum* and *Moschus*) and *Mel*. All constituents of the formulation will comply with *Korean Pharmacopoeia* standards [40]. A placebo will be manufactured by the same pharmaceutical company and will be comprised of corn starch as the main ingredient, as well as small amounts of *Dioscoreae rhizoma*, *Poria sclerotium*, *Mel*, a colouring agent, and a flavouring agent. Each pill will weigh 3.75 g and the amounts and sources of each component of the formulation are shown in Table 2. The participants will take GJD or placebo twice a day 30 min before breakfast and dinner, a total of six times during hospitalisation.

**Table 2:** Composition of *Gongjin-dan*

Herbal name		Scientific name (local name)	Quantity (g/pill)	Place of origin
Herbs	*Corni fructus*	*Cornus officinalis* Siebold et Zucc. (山茱萸)	0.9	Korea
	Korean angelica	*Angelica gigas* Nakai (當歸)	0.9	Korea
	*Ginseng radix*	*Panax ginseng* C.A. Meyer (人蔘)	0.9	Korea
Animal derives	*Moschus*	*Moschus moschiferus* L. (麝香)	0.15	Russia
	*Cornus cervi parvum*	*Cervus nippon* Temminck (鹿茸)	0.9	Russia
Diluting agents	*Mel*	*Apis indica* Radoszkowski (蜂蜜)		Korea
	*Aurum*	Gold (金)		Korea

During the study period, subjects will not be allowed to receive other fatigue-related treatments such as dietary supplements or alternatives. A practitioner will check to ensure that participants have taken the GJD or placebo immediately after administration and will collect empty bottles in order to confirm that all doses have been taken.

### Outcomes

Detailed outcome measurement time points are provided in Table 1.

#### Primary outcome

The primary outcome of this study is the participants’ serum cortisol levels. The effect of GJD on serum cortisol will be assessed by observing differences in cortisol levels before (day 2) and after (day 4) administration with GJD or placebo dosing after fatigue has been induced by sleep deprivation in healthy subjects. Considering circadian rhythms, cortisol will be measured at a specific time daily. Serum cortisol levels will be measured on an empty stomach 30 min after waking, and subjects will not eat foods that might affect cortisol levels, such as those high in sugar, fat, or protein for 12 h before the first administration of the investigational drug or placebo [12, 13].

#### Secondary outcome

Salivary cortisol levels, the secondary outcome of this study, will be measured once at admission on day 1 at 21:00, and three times daily on days 2–4; for example, cortisol will be measured within 30 min of waking, at 12:00, and at 21:00. Subjects will not eat within approximately 1 h or brush their teeth within 10 min before saliva collection in order to avoid contamination.

Blood samples for analysis of epinephrine, norepinephrine, oxidative stress-related biomarkers (reactive oxygen species (ROS), nitric oxide (NO), malondialdehyde (MDA), protein carbonyl, glutathione (GSH), GSH reductase (GSH-Rx), superoxide dismutase (SOD), catalase, and total antioxidant capacity (TAC)), homocysteine, and immunological factors (tumour necrosis factor (TNF)-α, interferon (IFN)-γ, interleukin (IL)-2, IL-10, IL-12, T cells, B cells, and natural killer (NK) cells) will be collected at the same time as the serum cortisol samples. Complete blood counts will be conducted to quantify the populations of T cells, B cells, and NK cells during phase II, day 2.

A self-reported questionnaire validated for each purpose and modified to a Korean version will be utilised to measure perceived fatigue. The FSS will be used to measure overall fatigue status [14], the BFI for variation in daily fatigue [15, 16], and the LSEQ for variation in sleep aspects [17]. The nine-item FSS is a self-reporting questionnaire developed by Krupp et al. [14] and serves to evaluate fatigue during the past week using statements that the participants must scale from 1 to 7 in terms of degree of agreement. The BFI is a self-reporting questionnaire developed by Mendoza et al. [15] to evaluate fatigue in patients with cancer. This nine-item questionnaire assesses the degree of fatigue at the present moment and for the past 24 h, as well as the impact of fatigue on daily life, on a scale of 0 to 10. The LSEQ consists of 10 items, each of which is measured on the Visual Analogue Scale (VAS); each item contains opposite statements at both extremes. The LSEQ involves four domains related to sleep: ease of initiating sleep (questions 1, 2, and 3), quality of sleep (questions 4 and 5), ease of waking (questions 6 and 7), and behaviour after waking (questions 8, 9, and 10). To complement the results of the BFI-K and the KMLSEQ, a daily sleep and fatigue status questionnaire developed by our research team will also be completed by the participants. This checks daily sleep and fatigue intuitively on the VAS and is structured so that participants can spontaneously describe a variety of their own symptoms.

In the afternoon of days 1–4, HRV testing will be performed to study variations in the autonomic nervous system responses relevant to fatigue, where HRV is measured as variations in the beat-to-beat intervals; R is a point corresponding to the peak of the QRS complex of the electrocardiogram (ECG) wave and RR is the interval between successive Rs. For HRV assessments, 5 min of ECG signalling will be measured and calculated using a Neo Dinamika system (MR Co. Ltd., Gyeonggi-do, Republic of Korea). The HRV measurements will be taken after participants rest for 10 min, and the subjects will be seated comfortably in a chair in a quiet and bright room at 20–25 °C for the assessment. The equipment will be connected to the medial side of both wrists and ankles as well as to the insides of both wrists and the right ankle. Rhythm parameters of the HRV include pulse rate, vegetative balance index, rhythm vegetative factor, regulation process adequacy index, and tension index. Vegetative regulation parameters of the HRV include regulation level and regulation reserves. Statistical analysis of the HRV includes the RRNN (mean of normal-to-normal (NN) intervals, which is all intervals between adjacent QRS complexes); the SDNN (standard deviation of NN intervals); CV (coefficient variation); the RMSSD (the root mean square of successive NN interval differences); the NN50 (the number of interval differences of successive NN intervals greater than 50 ms); and the pNN50 (the proportion of NN50 divided by total number of the mean of all normal NN intervals). Spectral analysis includes HF (high frequency, 0.15–0.4 Hz), LF (low frequency, 0.04–0.15 Hz), VLF (very low frequency, 0.0033 to 0.04 Hz), normalised HF, normalised LF, LF/HF ratio and TP (total power).

### Laboratory analysis

#### Determining stress hormones

Serum and salivary levels of cortisol as well as serum levels of norepinephrine and epinephrine will be assessed using enzyme-linked immunosorbent assay (ELISA) kits (LDN GmbH & Co., Nordhorn, Germany) according to the manufacturer’s protocol.

#### Determining oxidant biomarker levels in serum

Total serum levels of ROS will be determined according to the method described by Hayashi and colleagues [18]. Serum NO levels will be determined using the Griess method [19] and serum lipid peroxide levels will be determined using thiobarbituric acid (TBA) reactive substances as previously described [20]. The serum levels of protein carbonyl will be measured using a commercial ELISA kit (Cell Biolabs Inc., San Diego, CA, USA) and serum homocysteine levels will be analysed using an auto-analyser (AU400; Olympus, Tokyo, Japan).

#### Determining antioxidant biomarker levels in serum

Total antioxidant capacity (TAC) will be determined using a previously described method [21]. The serum levels of total glutathione (GSH) and GSH reductase (GSH-Rx) will be determined using commercial kits (Abcam, Cambridge, MA, USA) and SOD activity in the serum will be determined using an SOD assay kit (Dojindo Laboratories, Kumamoto, Japan) according to the manufacturer’s protocol. Serum catalase activities will be assayed as previously described [22].

#### Determining immune-related cytokine levels in serum

Serum levels of immune-related cytokines will be measured using commercial ELISA kits for TNF-α, IFN-γ as well as IL-2, IL-10, and IL-12 (BD Biosciences, San Jose, CA, USA). Absorbance will be measured with a spectrophotometer (Molecular Devices, Sunnyvale, CA, USA).

#### Flow cytometry and haematological analysis

Lymphocyte subclasses in the peripheral blood will be analysed using a BD Multitest IMK kit with a FACSCalibur instrument and Cell Quest Pro software (BD Biosciences, San Jose, CA, USA). Complete blood counts will be obtained using a Hemavet analyser (CDC Technologies, Dayton, OH, USA). T cells (CD3+), B cells (CD19+), and NK cells (CD3–/CD16+/CD56+) will be quantified as a percentage of the total number of lymphocytes.

### Randomisation and allocation concealment

Randomisation codes will be generated through block randomisation in a 1:1 code for groups A (phase I: GJD, phase II: placebo) and B (phase I: placebo, phase II: GJD). For the generation of a randomisation code, Random Allocation Software program v2.0.0 (M. Saghaei, MD, Department of Anaesthesia, Isfahan University of Medical Sciences, Isfahan, Iran) will be used.

### Blinding

A researcher who is not involved in recruitment or assessment will assign the clinical trial subjects identification numbers based on the randomisation code in order to avoid identifying groups. The number will be written on a piece of paper, put in a sealed envelope so that it is not visible, and stored in a double-locked cabinet. A researcher who is not involved in the generation of randomisation codes will open the envelopes sequentially and assign a clinical trial subject to each identification number. The opened envelope will also be stored separately in a double-locked cabinet. The clinical trial subjects will be informed that different medicines are given in phase I and phase II, but will not be told in which phase the therapeutic medicine is given. The process of attaching the randomisation code after manufacturing and packaging of GJD and placebo will be conducted by a researcher who is not involved in assessments to avoid breaking the blind. A researcher will provide medication immediately before each administration time and will check whether or not the subject takes the medication to avoid exchanging medicines between subjects during hospitalisation. To evaluate whether blinding of clinical trial subjects has been properly conducted, an assessment of blinding will be performed using a self-reported questionnaire developed by our research team at the end of the trial, at which point the subjects will be asked in which phase they perceived to have received the medication (as opposed to the placebo).

### Sample size

No previous clinical trials for serum cortisol levels from which sample size could be calculated have been reported; however, we consider a sample size of 24 to be a practical number for detection of clinically important differences between treatment and control groups. This number of subjects (12 per group) is the recommended minimum number per group for pilot studies [23]. We judged that this pilot study recommendation is applicable to feasibility study described here. Based on the results of this study, pilot trials will be needed in order to calculate appropriate sample size.

### Statistical analysis

An independent statistician who will be blinded to the randomised allocation for participants will carry out the statistical analyses. Measured variables including primary and secondary outcomes will be assessed using the full analysis set (FAS) based on intention-to-treat (ITT) principles. The per-protocol (PP) analysis set will be used for the sensitivity analysis. The baseline characteristics for both randomly allocated sequence groups are summarised by means and standard deviations for the continuous variables if satisfying the normal assumption, or median and interquartile range for nonnormal data. Normality will be tested by using the Shapiro-Wilk test. Frequencies and percentages will also be presented for categorical variables. The baseline difference between both sequence groups will be assessed using an independent two-sample *t* test or a Wilcoxon’s rank sum test for continuous variables, and the chi-squared test or Fisher’s exact test will be used to verify the randomised allocation for categorical variables.

Changes in serum cortisol levels between the baseline (day 2) and after treatment with GJD or placebo will be calculated to assess the primary outcome according to the following equations:

$$ {C}_{GJD}={X}_{GJD(E)}-{X}_{GJD(BL)} $$

$$ {C}_{PL}={X}_{PL(E)}-{X}_{PL(BL)} $$

where *C* is the change in serum cortisol level, *X* is the serum cortisol level, *E* is the end point, *BL* is the baseline, *GJD* is *Gongjin*-*dan* treatment, and *PL* is placebo.

The primary outcome analysis (*Δ = D*_*GJD*_*– D*_*PL*_) will be performed in two stages. First, a linear mixed effect model will be applied to assess the effect of treatment, sequence, and period with the random effect for subjects. The carryover effect due to preceding the treatment stage will then be estimated by testing the effect of sequence in the model. The linear mixed model will be modified by confounding the sequence effect to the error term if the carryover effect is not significant. Otherwise, the model will be rebuilt based on data collected in the first phase of the study. An analysis of variance (ANOVA) table for the model will be provided and least square means and their standard errors (95 % confidence intervals) will be reported for each treatment. Second, if the structure of data is balanced and the carryover effect is invalid, simple paired two-sample *t* tests or Mann Whitney *U* tests will be performed to evaluate the effect of GJD. The results will be shown as means and standard deviations for each treatment group and mean differences between the two treatments (95 % confidence interval) will be reported.

The analysis of secondary outcomes will be focussed on changes of measures within each treatment group from the baseline to the end of study. Again, the linear mixed effect model will be applied. The level of significance will be set to 0.05 (two-tailed) and all analyses will be performed with PROC MIXED and PROC GLM in SAS software, version 9.4 (SAS Institute Inc., Cary, NC, USA).

### Data and safety monitoring

Regular monitoring will be conducted to ensure quality control of the data according to the planned protocol and standard operating procedures. Monitors blinded to the allocation information will evaluate whether the recruitment procedures are correctly performed and whether the data are adequately recorded according to the protocol in the case report forms. If there are necessary changes to the study methods such as changes to the eligibility criteria, treatment regimens, or follow-up periods, the investigators will discuss these potential changes with independent researchers and statisticians. In the event that severe adverse events and crucial issues occur, the investigators will determine whether these issues are acceptable or if the trial should be amended or ended.

### Safety and adverse events

For the safety of study participants, complete blood counts, liver function tests, and urine analyses will be performed at the screening phase and the end of phases I and II, and vital signs will be checked once daily. Contact information will be provided to all participants for reporting of adverse events at any time. Practitioners will also check for any expected or unexpected adverse events every evening of the hospitalisation periods. Adverse events known to be related to GJD treatment include gastric discomfort, anorexia, nausea, diarrhoea, rash, urticaria due to drug-related allergic reaction, minimal-change disease, and acute focal tubulointerstitial nephritis [24]. Any adverse events reported by participants will be recorded for a causal relationship in the ‘Adverse event record table’. A causal relationship between the GJD or placebo treatment and adverse events will be evaluated using a six-grade scale (1 = definitely related, 2 = probably related, 3 = possibly related, 4 = probably not related, 5 = definitely not related, and 6 = unknown) and the seriousness of any such events will be scored using a three-point scale (1 = mild, 2 = moderate, and 3 = severe). Any adverse events will be reported in accordance with the regulations of the Institutional Review Board.

### Ethics

This study complies with the principles of the Declaration of Helsinki and Good Clinical Practice guidelines. The protocol has been reviewed and approved by the Institutional Review Board of the Daejeon Korean Medicine Hospital of Daejeon University (DJOMC-127-1) and is registered with the national clinical trial registry Clinical Research Information Service, which is a primary registry of the World Health Organisation International Clinical Trials Registry Platform (http://apps.who.int/trialsearch/Default.aspx; CRIS registration No. KCT0001681). Any protocol modifications will be approved by the Institutional Review Board of the Daejeon Korean Medicine Hospital of Daejeon University.

Written informed consent will be obtained from all study participants prior to enrolment into the study and participants will be given the option to decline to participate or withdraw at any time without disadvantage.

## Discussion

Deciding on the appropriate primary outcome of a trial is one of the challenges of trial design. The primary outcome in this study protocol is serum cortisol level measured at 30 min after waking. Cortisol is closely related to fatigue and blunted cortisol levels are primarily observed in patients with fatigue [25–27]. Although fatigue is a subjective symptom, cortisol levels were selected as the primary variable over the questionnaire-derived results.

Cortisol should be measured at the same time every day due to circadian rhythm. Cortisol levels may, moreover, be affected by dietary supplements [28], caffeine [29], and intensive exercise [30], as well as foods with a high sugar, fat, or protein content [12, 13]. The crossover method and participant hospitalisation were implemented in this study to minimise confounding factors and other possible bias [31].

To determine perceived symptoms and to compare these with objective outcomes, the results of self-reported questionnaires including the FSS-K, BFI-K, KMLSEQ, and daily fatigue and sleep evaluation questionnaires will be evaluated.

Epinephrine, norepinephrine, and HRV are related to the autonomic nervous system and as such are stimulated as a response to stress in the human body. Most individuals experiencing fatigue have hyperactive sympathetic nerves and hypoactive parasympathetic nerves [32, 33], and variations in autonomic nerve activity in the fatigue state can be observed as changes in serum concentrations of epinephrine and norepinephrine as well as in HRV [34].

In comparisons of normal individuals to those experiencing fatigue, it has been reported that circulating oxidative stress increases with fatigue, but that antioxidant levels decrease [35–37]. Immunity disturbances have been researched for potential involvement in chronic fatigue syndrome and analysis of immunological factors are, therefore, also included in this study [38, 39].

This is a feasibility study and is, thus, not powered to determine treatment effectiveness. As appropriate sample size could not be determined due to a lack of similar previous studies, the minimal sample size considered clinically significant was defined. The results of this study may, therefore, affect generalisability in other environments. Moreover, because fatigue will be artificially induced in healthy people in this study, the findings would not represent chronic fatigue, which is more clinically relevant. Further study with exact sample sizes in patients complaining of chronic fatigue would be necessary and could be designed based on the results from this study. This study protocol represents a unique opportunity to enhance our understanding of fatigue and the effects GJD on fatigue in terms of endocrine and immunological mechanisms and may, therefore, serve to improve future treatment strategies for chronic fatigue.

### Trial status

Recruitment for this trial opened in September 2015 and will close in December 2015. At the time of manuscript submission, the trial was in the recruitment phase.

## Abbreviations

ALT: Alanine aminotransferase
ANOVA: Analysis of variance
AST: Aspartate aminotransferase
BFI-K: Brief Fatigue Inventory-Korean version
CAM: Complementary and alternative medicine
CV: Coefficient variation
ECG: Electrocardiogram
ELISA: Enzyme-linked immunosorbent assay
FAS: Full analysis set
FSS-K: the Fatigue Severity Scale-Korean version
GJD: *Gongjin*-*dan*
GSH: Glutathione
GSH-Rx: Glutathione reductase
HF: High frequency
HRV: Heart rate variability
IL: Interleukin
INF-γ: Interferon gamma
ITT: Intention-to-treat
KMLSEQ: Korean Version of the Modified Leeds Sleep Evaluation Questionnaire
LF: Low frequency
MDA: Malondialdehyde
MFDS: Ministry of Food and Drug Safety
NK cells: Natural killer cells
NN intervals: Normal-to-normal intervals
NN50: Number of interval differences of successive NN intervals greater than 50 ms
NO: Nitric oxide
pNN50: Proportion of NN50 divided by total number of the mean of all normal NN intervals
PP: Per-protocol
PSQI-K: Pittsburgh Sleep Quality Index-Korean version
RMSSD: Root mean square of successive NN interval differences
ROS: Reactive oxygen species
RRNN: Mean of normal-to-normal intervals, which is all intervals between adjacent QRS complexes
SDNN: Standard deviation of NN intervals
SOD: Superoxide dismutase
TAC: Total antioxidant capacity
TNF-α: Tumour necrosis factor alpha
TP: Total power
VAS: Visual Analogue Scale
VLF: Very low frequency
